# The Effect of Oxidative Phosphorylation on Cancer Drug Resistance

**DOI:** 10.3390/cancers15010062

**Published:** 2022-12-22

**Authors:** Ziyi Zhao, Yong Mei, Ziyang Wang, Weiling He

**Affiliations:** 1 Department of Gastrointestinal Surgery, The First Affiliated Hospital, Sun Yat-Sen University, Guangzhou 510080, China; 2 Center for Precision Medicine, Sun Yat-Sen University, Guangzhou 510080, China

**Keywords:** oxidative phosphorylation, resistance, metabolism, glycolysis, cancer immunity

## Abstract

**Simple Summary:**

Drug therapy is an important treatment for cancer patients; however, drug resistance severely affects the survival time and quality of life of cancer patients. Oxidative phosphorylation (OXHPOS) is an important metabolic process in cells that drives cancer drug resistance and exerts a significant influence on responses to anticancer therapy. Targeting OXPHOS can specifically eliminate cancer stem cells and delay the acquisition of drug resistance. Hence, OXPHOS has become a novel pharmacological target in cancer treatment. OXPHOS inhibitors in combination with conventional therapies have significantly increased the efficacy of treatments and attenuated resistance to anticancer drugs.

**Abstract:**

Recent studies have shown that oxidative phosphorylation (OXPHOS) is a target for the effective attenuation of cancer drug resistance. OXPHOS inhibitors can improve treatment responses to anticancer therapy in certain cancers, such as melanomas, lymphomas, colon cancers, leukemias and pancreatic ductal adenocarcinoma (PDAC). However, the effect of OXPHOS on cancer drug resistance is complex and associated with cell types in the tumor microenvironment (TME). Cancer cells universally promote OXPHOS activity through the activation of various signaling pathways, and this activity is required for resistance to cancer therapy. Resistant cancer cells are prevalent among cancer stem cells (CSCs), for which the main metabolic phenotype is increased OXPHOS. CSCs depend on OXPHOS to survive targeting by anticancer drugs and can be selectively eradicated by OXPHOS inhibitors. In contrast to that in cancer cells, mitochondrial OXPHOS is significantly downregulated in tumor-infiltrating T cells, impairing antitumor immunity. In this review, we summarize novel research showing the effect of OXPHOS on cancer drug resistance, thereby explaining how this metabolic process plays a dual role in cancer progression. We highlight the underlying mechanisms of metabolic reprogramming in cancer cells, as it is vital for discovering new drug targets.

## 1. Introduction

In 1924, Otto Warburg found that cancer cells increased glucose uptake and augmented glycolysis to elevate ATP production even when ample oxygen was available for oxidative phosphorylation (OXHPOS), and this phenomenon was termed the Warburg effect. Warburg thought that cancer cells showed defects in mitochondrial OXPHOS [1,2]. However, multiple investigators later found that cancer cells carry functional mitochondria and show upregulated OXPHOS activity following treatment with anticancer drugs [3,4,5]. In 2000, researchers studying certain OXPHOS disorders discovered mutations in the structural OXPHOS genes in hereditary paraganglioma (PGL), a vascularized tumor in the paraganglia [6,7]. This work indicated that carcinogenesis is probably associated with mitochondrial gene mutations. Cancer cells with mitochondrial gene mutations exhibit growth advantages during tumorigenesis [8]. Although mitochondrial function has been erroneously assumed to be inessential for tumorigenesis, mitochondrial metabolism has been a recurring target in cancer therapy [9,10,11]. Accumulating evidence suggests that in certain cancers, such as lymphomas and endometrial carcinoma, OXPHOS is upregulated, and OXPHOS inhibitors can therefore exert a repressive effect on these cancers [3,12].

Drug resistance severely affects the survival time and quality of life of cancer patients. Moreover, drug resistance is mediated by complex mechanisms, such as drug efflux, the TME, and overexpression of oncogenes. Recent data have demonstrated that mitochondrial OXPHOS drives cancer drug resistance and exerts a significant influence on responses to anticancer therapy [13,14,15]. OXPHOS is required for cancer cells to acquire drug resistance in various cancers. Cancer cells show enhanced mitochondrial OXPHOS mediated by the activation of several oncogenic signaling pathways. In hematologic malignancies, cancer stem cells (CSCs) display metabolic remodeling and increased OXPHOS activity, which enables them to adjust to the fluctuating TME. CSCs are thought to be enriched and display chemotherapy resistance after exposure to antitumor drugs, inducing cancer relapse. Targeting OXPHOS can specifically eliminate CSCs and delay the acquisition of drug resistance [16,17,18,19,20]. Hence, OXPHOS has become a novel pharmacological target in cancer treatment. OXPHOS inhibitors in combination with conventional therapies have significantly increased the efficacy of treatments and attenuated resistance to anticancer drugs [18,21,22,23].

## 2. The OXPHOS Metabolic Pathway

**Figure 1:**
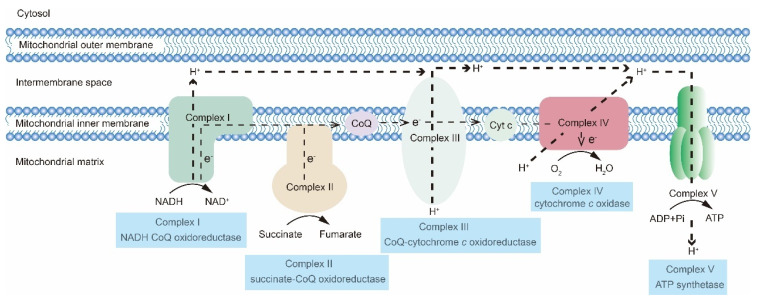
Mitochondrial OXPHOS system. Electrons are transferred via NADH to Complex I and then transported to CoQ. Flavin-containing enzyme complexes can directly deliver electrons to CoQ. CoQ transfers electrons to Complex IV through Complex III and Cyt c. In this step, oxygen is reduced to water. Complexes I, III, and IV pump H^+^ from the mitochondrial matrix into the intermembrane space, which generates a proton gradient. Eventually, Complex V leverages this proton gradient to produce ATP.

The OXPHOS system reduces the oxygen level and produces ATP through a series of protein complexes, together termed the electron transport chain (ETC), which is embedded in the mitochondrial inner membrane. The ETC consists of Complexes I–V and two electron carriers, cytochrome c (Cyt c) and coenzyme Q (CoQ) [24,25]. The OXPHOS system is controlled by both the nuclear and mitochondrial genomes. The mitochondrial genome (mtDNA) encodes 13 protein subunits of the OXPHOS system, and the nuclear genome encodes at least 70 OXPHOS subunits [26]. In the OXPHOS process, NADH transfers electrons to Complex Ⅰ. Electrons are subsequently transported to CoQ. Notably, flavin-containing enzyme complexes can directly deliver electrons to CoQ. CoQ transfers electrons to Complex IV through Complex III and Cyt c, and in this step, water is generated due to oxygen reduction. Complexes I, III, and IV pump H^+^ from the mitochondrial matrix into the intermembrane space, which generates a proton gradient. Eventually, Complex V leverages this proton gradient to produce ATP (Figure 1).

OXPHOS genes are also regulated by several nuclear genes through various mechanisms [27,28,29]. PGC-1 is a transcriptional coactivator that promotes mitochondrial biogenesis and respiration by inducing uncoupling protein-2 (UCP-2). In addition, PGC-1 upregulates the expression of nuclear respiratory factor (NRF) 1 and 2, both of which are associated with the mitochondrial bioenergetic machinery [30,31].

Defects in OXPHOS genes lead to a wide variety of mitochondrial disorders [32,33,34]. Leigh syndrome, a subcortical encephalopathy, is the most frequently diagnosed mitochondrial disorder [35,36,37]. The typical symptoms include optic atrophy, ataxia, and hypotonia. Leigh syndrome is caused by mutations in Complex I or Complex IV. Of note, researchers have identified some mutations in the SDHD gene in PGL patients. SDHD encodes a small subunit of cytochrome b, which is a component of the mitochondrial respiratory chain [7]. This study suggests that mitochondria may play important roles in tumorigenesis.

## 3. Resistant Cancer Cells Display High OXPHOS Activity Levels

**Figure 2:**
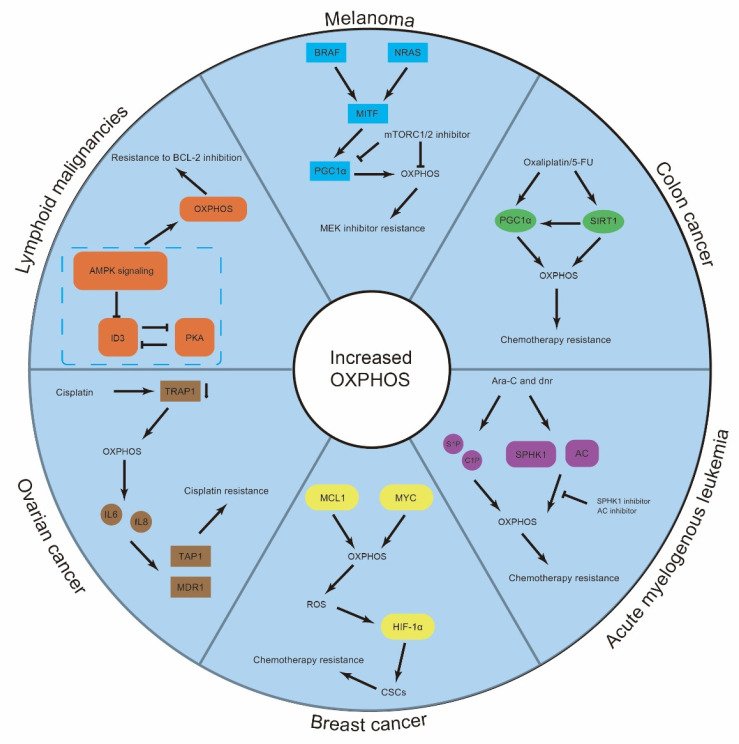
Resistant cancer cells display high OXPHOS activity. Activation of various oncogenic signaling pathways contributes to cancer drug resistance by upregulating OXPHOS activity in certain cancers, including breast cancer, ovarian cancer, and melanoma.

Mitochondrial mutations in certain human tumors may contribute to cell proliferation advantages in tumorigenesis [8]. Drug-sensitive cancer cells promote glucose utilization and undergo enhanced glycolysis, promoting their rapid proliferation. Resistant cancer cells exhibit reprogrammed metabolism, which drives an energy metabolism shift, mediated via the expression of certain oncogenes, toward OXPHOS [38] (Figure 2).

### 3.1. OXPHOS in the Drug Resistance of Hematologic Malignancies

The transition of energy metabolism to OXPHOS is common in hematologic malignancies. Ceramide can target mitochondria and contribute to cell apoptosis [39,40]. Resistant cells in acute myelogenous leukemia (AML) activate sphingosine kinase 1 (SPHK1) and acid ceramidase (AC), and they display increased OXPHOS, promoting drug resistance. The SPHK1 inhibitor SK1-i and AC inhibitor SACLAC partially decrease OXPHOS activity [41].

Lymphoma cells activate the AMPK signaling pathway to inhibit the lymphoid transcription regulator ID3, which leads to the upregulation of the PKA subunit *PRKAR2B*. In turn, *PRKAR2B* overexpression results in reduced ID3 protein levels. The pathway consisting of ID3, AMPK, and PKA increases mitochondrial OXPHOS, which is involved in B-cell lymphoma 2 (BCL-2) inhibitor resistance [42]. Twenty percent of AML patients harbor IDH mutations, including IDH2 R140 and IDH1 R132 [43]. AML patients with IDH mutations display enhanced OXPHOS, which is induced by an increase in Complex I activity. IDH1 mutant inhibitors fail to decrease mitochondrial respiration due to PGC1α activation and Akt inhibition. OXPHOS inhibitors improve the response to IDH1 mutant inhibitors in AML [44]. The internal tandem duplication (ITD) of the tyrosine kinase receptor FLT3 (*FLT3*-ITD) is a chromosomal aberration, occurring in 30% of AML patients [45,46]. FLT3 inhibitor gilteritinib is used as frontline treatment or AML patients. *FLT3*-ITD AML cells develop resistance to gilteritinib by switching from glycolysis to OXPHOS. Dihydroorotate dehydrogenase (DHODH), cyclin-dependent kinase 9 (CDK9), or protein arginine N-methyltransferase 5 (PRMT5) inhibition decreases OXPHOS activity and sensitizes cells to gilteritinib treatment [47].

### 3.2. OXPHOS in Drug Resistance of Solid Tumors

In solid tumors, resistance to MEK inhibitors in human melanomas with BRAF and NRAS mutations is mediated by high OXPHOS activity, which is attenuated by mTORC1/2 inhibitors. Mechanistically, human melanomas with BRAF and NRAS mutations facilitate MITF expression and upregulate the level of the transcriptional coactivator PGC1α. An mTORC1/2 inhibitor promotes MITF translocation from the nucleus to the cytoplasm, inhibiting PGC1α expression and OXPHOS activity [48]. PGC1α can synergize with the histone deacetylase sirtuin 1 (SIRT1) to promote resistance to chemotherapy; this synergistic effect is mediated by increased OXPHOS activities in colon cancer. Targeting the SIRT1/PGC1a pathway inhibits mitochondrial OXPHOS, enhancing drug efficacy [49].

Breast CSCs (BCSCs) can overexpress MYC and MCL1 and thus survive cytotoxic chemotherapy in triple-negative breast cancer (TNBC). MYC and MCL1 upregulate OXPHOS activity, which in turn increases HIF-1α expression to promote CSC enrichment [50]. BAY-876 is the first highly selective GLUT1 inhibitor that increases esophageal squamous cell carcinoma (ESCC) cell sensitivity to cisplatin [51,52]. Some TNBC cell lines exhibit resistance to BAY-876 through high levels of OXPHOS. On the other hand, BAY-876-sensitive TNBC cells display low OXPHOS rate and increased glycolysis [53]. Similarly, ovarian cancer (OC) cells increased oxidative metabolism to drive cisplatin resistance via the downregulation of tumor necrosis factor receptor-associated protein 1 (TRAP1). TRAP1-mediated metabolic reprogramming significantly induces the upregulation of two members of the multidrug resistance protein family, TAP1 and MDR1, activating IL signaling and stimulating IL6 expression [54]. Histone deacetylase (HDAC) inhibitors have been used in the treatment of hematologic malignancies [55]. However, HDAC inhibitors do not have a therapeutic effect on solid tumors [56]. In glioblastoma (GBM), HDAC inhibitors result in high OXPHOS activity mediated by elevated fatty acid oxidation (FAO). HDAC inhibitors increase the expression of PGC1α to enhance OXPHOS, which is dependent on c-Myc [57].

### 3.3. ROS Levels in Cancer Cells and OXPHOS

Both an oxidative state and a glycolytic state are hallmarks of cancer cells, which display a different metabolic signature than normal cells [58,59,60]. This difference results from increased reactive oxygen species (ROS) levels and activated oncogenic pathways, such as the c-SRC, MYC and RAS pathways, in cancer cells. A specific hybrid state of cancer cells promotes their metabolic plasticity [61]. High OXPHOS activity in resistant cancer cells increases the levels of ROS. Upon reaching certain levels, ROS promote cancer cell proliferation [62]. The ERK1/2 MAPK pathway can mediate cell proliferation when ROS levels are low. Researchers have shown that ROS are mainly generated from the Qo site in Complex III. Mitochondrial ROS mediate Kras-induced cell proliferation and carcinogenesis by inhibiting ERK1/2 MAPK pathway activity [63].

However, excess ROS contribute to cancer cell death. Chemoresistant cancer cells show enhanced ROS-scavenging system activity and decreased intracellular ROS levels, enabling them to survive chemotherapy. Cisplatin-resistant ovarian carcinoma cells elevate the pentose phosphate pathway (PPP), which promotes GSH production to maintain redox homeostasis and contribute to cisplatin resistance [64,65]. Peroxiredoxin 3 (Prx3) is a ROS detoxification gene. FoxM1 promotes Prx3 expression and decreases ROS levels in gastric CSCs critical for chemoresistance [66]. Researchers have found that the leukemia cell populations with decreased ROS levels are enriched with leukemia stem cells (LSCs) and that LSCs depend on OXPHOS. This cell subpopulation exhibits upregulated BCL-2, which is associated with the inhibition of the mitochondria-initiated pro-apoptotic pathway. BCL-2 inhibitors selectively eradicate LSCs by increasing ROS production and inhibiting OXPHOS activity [17]. Targeting the ROS-scavenging system probably reverses chemotherapy resistance via ROS-induced cell death. Protoporphyrin is a photosensitizer used in photodynamic therapy. Under laser irradiation, protoporphyrin can produce hydroxyl anions and induce cancer cell death [67].

## 4. CSCs Undergo a Metabolic Transition between OXPHOS and Glycolysis

### 4.1. CSC Metabolism Remodeling and Promotion of Mitochondrial OXPHOS

CSCs constitute a subpopulation of self-renewing cells with differentiation potential. These cells give rise to resistance to conventional therapies, contributing to a decrease in the survival time of cancer patients [68,69,70].

CSCs activate DNA repair pathways and protect DNA from chemotherapy [71]. Translesion DNA synthesis (TLS) is a specialized DNA damage tolerance pathway mediated by DNA polymerases of the Y-family and the B-family [72]. Ovarian CSCs promote the expression of DNA polymerase η (Pol η), a member of the DNA polymerase Y-family. Pol η mediates cisplatin resistance in ovarian CSCs by inducing TLS [73]. On the other hand, CSCs show therapy resistance by increasing drug efflux activity [71]. ATP-binding cassette transporters have ATP-binding domains, which are associated with multidrug resistance [74]. ATP-binding cassette subfamily B member 1 (ABCB1) is elevated in CSCs and contributes to MET inhibitor resistance in non-small cell lung cancer (NSCLC) [75]. Compared to non-CSCs, CSCs have been shown to have a distinct metabolic status in various cancers. Metabolism reprogramming of CSCs is required for supporting stemness properties [76,77]. The homeobox transcription factor NANOG maintains self-renewal and pluripotency of embryonic cells in human development. However, overexpression of NANOG leads to chemotherapy resistance in CSCs by regulating metabolic pathways [78]. CSCs activate FAO to induce sorafenib resistance, which is a kinase inhibitor used for hepatocellular carcinoma (HCC). This resistance is dependent on the overexpression of NANOG. Inhibition of FAO sensitizes CSCs to sorafenib [79]. Cyclophosphamide is an alkylating antineoplastic agent that is used for the treatment of various cancers [80]. Aldehyde dehydrogenase 1 (ALDH1) may mediate cellular resistance to cyclophosphamide [81]. Colon CSCs can secrete high levels of ALDH1 and neutralize the cytotoxicity of maphosphamide, an active form of cyclophosphamide [82]. Anticancer drugs can enrich CSCs, remodel their metabolism and promote mitochondrial OXPHOS [16,18,20].

The tyrosine kinase inhibitor (TKI) imatinib has become a pillar of chronic myeloid leukemia (CML) treatment. However, LSCs acquire resistance that results in disease relapse. In LSCs, oxidative metabolism is upregulated, which is crucial for surviving anticancer drugs. Therefore, imatinib in combination with the mitochondrial translation inhibitor tigecycline can significantly eliminate LSCs and delay resistance to targeted therapy [16]. The DNA hypomethylating agent azacytidine, in combination with the BCL-2 inhibitor venetoclax, suppresses Complex II activity by reducing sdhA glutathionylation, which inhibits OXPHOS activity in acute myeloid leukemia stem cells (AML LSCs). Azacitidine + venetoclax selectively eradicates LSCs and induces durable and deep responses in AML patients [18]. However, in relapsed/refractory (R/R) AML patients, this treatment displays less efficacy. Azacitidine+ venetoclax cannot eliminate relapsed LSCs that promote nicotinamide metabolism to induce OXPHOS. Nicotinamide phosphoribosyltransferase (NAMPT) inhibitors specifically target relapsed LSCs by reducing OXPHOS activity but do not affect normal hematopoietic stem cells [83].

Calcitonin receptor-like receptor (CALCRL) is involved in several processes, including receptor internalization and the G protein-coupled receptor signaling pathway. CALCRL inhibition impairs AML cell proliferation, decreases LSC enrichment and confers chemotherapy sensitivity to AML cells. Mechanistically, CALCRL induces mitochondrial OXPHOS required for BCL2 and E2F1 [84]. Bone marrow mesenchymal stem cells (BMSCs) are heterogeneous populations that contain various progenitor cells. BMSCs are associated with cell proliferation and immune regulation [85]. Nestin is a marker of neuroepithelial stem cells. BMSCs expressing nestin contribute to the chemoresistance of LSCs. Nestin BMSCs provide metabolic support to LSCs through OXPHOS and the TCA cycle [86]. NOTCH1 mutation is common in T-cell acute lymphoblastic leukemia (T-ALL), which is an aggressive hematologic malignancy [87,88]. The OXPHOS pathway is driven by oncogenic activation of NOTCH1 in T-ALL and plays a significant role in LSC function. NOTCH1 status affects LSC sensitivity to OXPHOS inhibitors. T-ALL cells with NOTCH1 mutations show a greater response to OXPHOS inhibition than NOTCH1 -wt cells [89].

Signal transducer and activator of transcription 3 (STAT3), a cytoplasmic transcription factor, is involved in various oncogenic signaling pathways [90]. STAT3 induces OXPHOS activity in LSCs. At the molecular level, STAT3 promotes MYC expression in AML; in turn, MYC regulates the transcription of SLC1A5, a neutral amino acid transporter gene. Inhibition of STAT3 can specifically eradicate AML CSCs and progenitor cells [91]. Similar to their effect on hematologic malignancies, CSCs increase OXPHOS activity in solid tumors. A subpopulation of PDAC cells survive mutated KRAS ablation. Resistant cells express cancer stem cell markers with OXPHOS activation and impaired glycolysis. In the treatment of OXPHOS inhibitors, resistant cells fail to upregulate glycolysis to compensate for decreased ATP production. OXPHOS inhibitors specifically eradicate resistant cells [20]. Colon CSCs exhibit increased ROS production and consume more oxygen than non-CSCs. Colon CSCs exhibit upregulation of the mitochondrial gene PRX3, which promotes mitochondrial function and maintains cancer stemness. FOXM1 binds to the PRX3 promoter to enhance its expression [92]. Deoxyguanosine kinase (DGUOK) is a mitochondrial deoxynucleoside kinase associated with the salvage of purine deoxynucleoside. DGUOK promotes CSC self-renewal in lung adenocarcinoma. At the molecular level, DGUOK facilitates Complex I activity to maintain mitochondrial OXPHOS, which regulates the AMPK-YAP1 pathway and maintains cancer cell stemness [93].

Notably, recent data demonstrate that chemotherapy-resistant cancer cells do not necessarily exhibit stem cell features, which still increase mitochondrial respiration. Cytarabine (Ara-C) is a nucleoside drug that is commonly used in the treatment of leukemia. Researchers have shown that Ara-C treatment cannot induce LSC enrichment, a result different from that of a previous study [94]. Ara-C-resistant cells increase OXPHOS activity by promoting fatty acid oxidation and overexpression of CD36 [95]. Researchers have also found that Ara-C leads to the upregulation of the ectonucleotidase CD39 in AML patients, which is related to poor prognosis in the clinic. Increased CD39 activity contributes to Ara-C resistance by promoting mitochondrial OXPHOS. Inhibition of CD39 activity inhibits the metabolic shift triggered by Ara-C and increases AML cell sensitivity to Ara-C in vitro [96].

### 4.2. Certain Drugs Cause Metabolic Transformation from OXPHOS to Glycolysis in CSCs

Although an increasing body of evidence shows that cancer therapy induces a metabolic shift toward OXPHOS, researchers have also found that the transition of the metabolic process from OXPHOS to glycolysis occurs in CSCs after treatment with certain antitumor drugs [76,97,98].

Metformin is a biguanide used to treat type 2 diabetes. Metformin inhibits mitochondrial complex I to decrease OXPHOS activity [99]. Metformin has been suggested to increase intracellular ROS levels and induce apoptosis in pancreatic CSCs. However, CSCs treated with metformin eventually develop resistance, contributing to tumor progression [100]. In hepatocellular carcinoma (HCC) cells, metformin can lead to increased OXPHOS. Mechanistically, TOMM34, a translocase in the outer mitochondrial membrane, interacts with ATP5B and maintains OXPHOS activity in HCC cells treated with metformin. The OXPHOS inhibitor gboxin sensitizes HCC to metformin by abrogating the interaction between TOMM34 and ATP5B [101].

Metformin-resistant pancreatic CSCs exhibit specific metabolic features, including downregulated OXPHOS and increased glycolytic activity. Mechanistically, upregulation of MYC inhibits PGC-1α expression in metformin-resistant CSCs by binding to its promoter. PGC-1α is associated with mitochondrial biogenesis and promotes OXPHOS. Interestingly, the mitochondrial ROS inducer menadione can reverse resistance to metformin in pancreatic CSCs. Menadione probably becomes a promising drug to attenuate the resistance of cancer cells to OXPHOS inhibitors [102].

Researchers have also found that pancreatic CSCs upregulate the expression of interferon-stimulated gene 15 (ISG15) and promote protein ISGylation. Loss of ISG15 impairs pancreatic CSC sphere formation capacity. ISG15 knockout contributes to reduced mitochondrial ISGylation and decreased OXPHOS. Importantly, loss of ISG15/ISGylation reverses pancreatic CSC resistance to metformin [103]. Toll-like receptor 4 (TLR4) signaling activates NANOG through E2F1 phosphorylation in HCC. NANOG impairs mitochondrial OXPHOS to reduce ROS production, which is associated with drug resistance. Mitochondrial metabolic signaling linked with NANOG maintains tumor-initiating stem-like cells (TICs). Upregulation of OXPHOS sensitizes TICs to the kinase inhibitor sorafenib in advanced HCC [79].

## 5. OXPHOS Plays a Dual Role in Cancer Immunity

### 5.1. OXPHOS Contributes to Immunotherapy Resistance

Immune checkpoint therapy has led to great successes in cancer treatment and can enhance T-cell responses to induce durable clinical responses for many kinds of cancers [104,105,106]. Targeting the programmed cell death protein-1 pathway (PD-1/PD-L1) and cytotoxic T lymphocyte–associated antigen-4 (CTLA-4) has led to important clinical advances in cancer therapy [107,108,109]. However, this treatment can elicit only tumor regression in a fraction of patients. Some patients do not respond; that is, they show primary resistance, while others develop acquired resistance after the initial response [110,111,112]. Metabolic reprogramming occurs in immune cells and cancer cells and contributes to immunotherapy resistance [113,114,115].

Melanoma cells resistant to anti-PD-1, anti-PD-L1 and anti-CTLA-4 immunotherapy show increased OXPHOS activity. In melanoma patients resistant to anti-PD-1+ anti-CTLA-4 immunotherapy, OXPHOS-related genes are upregulated. The OXPHOS inhibitor metformin fails to sensitize resistant melanoma cells to immunotherapy [116]. A PD-1-resistant murine tumor model showed greater oxidative metabolism than a PD-1-sensitive model of non-small cell lung cancer (NSCLC). IACS-010759 is a Complex I inhibitor and mediates apoptosis in AML dependent on OXPHOS [117]. IACS-010759 in combination with radiotherapy sensitized the PD-1-resistant model to anti-PD-1 agents and prolonged survival time. This combination treatment is being tested clinically [118].

### 5.2. OXPHOS Affects Certain Types of Immune Cells in The TME

**Figure 3:**
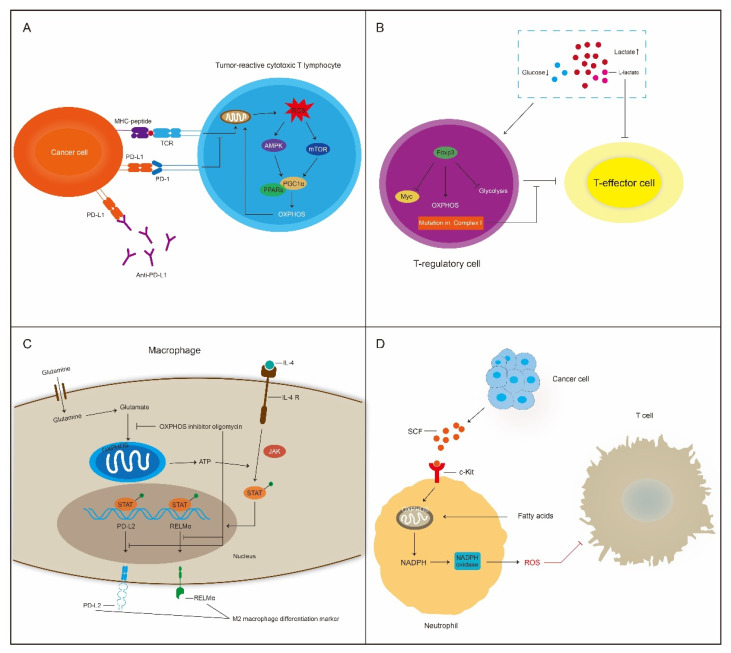
OXPHOS plays a dual role in cancer immunity. (**A**) Anti-PD-L1 agent promotes mitochondrial function in tumor-reactive cytotoxic T lymphocytes (CTLs). (**B**) The Foxp3-mediated metabolic phenotype of Tregs may result in resistance to anticancer immunotherapy. (**C**) OXPHOS is required for M2 macrophage differentiation. (**D**) Neutrophils may mediate immune suppression in the tumor microenvironment (TME).

The influence of OXPHOS in immune cells on immunotherapy resistance is related to certain types of immune cells in the TME [119] (Figure 3). Tumor-infiltrating T cells have reduced mitochondrial OXPHOS activity and display mitochondrial dysfunction. Nicotinamide riboside can improve the mitochondrial function of tumor-infiltrating T cells and enhance responsiveness to PD-1 blockade [120]. Decreased mitochondrial mass in tumor-infiltrating T cells is associated with high expression of immune inhibitory molecules in the TME; these proteins include Tim-3, LAG-3, and PD-1. Inhibition of PGC1α contributes to low OXPHOS activity, which is partially mediated by activation of the Akt signaling pathway. Tumor-infiltrating T cells enhance antitumor immunity through PGC1α overexpression [121]. Anti-PD-L1 agents promote the mitochondrial function of tumor-reactive cytotoxic T lymphocytes (CTLs), inducing high ROS generation. In turn, ROS can augment the T-cell-dependent antitumor activity of anti-PD-L1 agents. Mechanistically, ROS activate AMPK and mTOR signaling, which upregulates PGC1α expression. PGC1α, with its cofactor peroxisome proliferator-activated receptors (PPARs), mediates mitochondrial OXPHOS to enhance antitumor immunity. Hence, combinatorial therapy comprising PGC1α activators with anti-PD-L1 agents is a promising strategy to improve the efficacy of cancer immunotherapy [122].

T-regulatory cells (Tregs) constitute a subset of helper T cells and maintain immune equilibrium by inhibiting various immune cell activities [123]. Tregs facilitate OXPHOS in low-glucose and high-lactate environments; these effects are induced by the expression of forkhead box P3 (Foxp3). Treg suppressive function against T-effector (Teff) cells is significantly reduced by mutation in ETC complex I. The Foxp3-mediated metabolic phenotype of Tregs may result in resistance to anticancer immunotherapy [124]. However, the effect of OXPHOS on Treg suppressive activity is heterogeneous. Fatty acid binding protein 5(FABP5) is a lipid chaperone that promotes lipid uptake. Inhibition of FABP5 disrupts lipid metabolism, leading to reduced OXPHOS activity in Tregs. In contrast to previous studies, FABP5 inhibition facilitates type I IFN signaling and Treg suppressive activity [125].

Macrophages play an important role in inflammatory responses and tissue homeostasis [126,127]. Macrophages differentiate into two main subtypes: proinflammatory M1 macrophages and anti-inflammatory M2 macrophages. M1 macrophages induce the death of tumor cells and increase glycolysis. M2 macrophages mediate immune tolerance and recruit Tregs to promote immunotherapy resistance with the metabolic signature of high OXPHOS activity [128]. OXPHOS is required for M2 macrophage differentiation [129]. The OXPHOS inhibitor oligomycin significantly decreases the expression of PD-L2 and RELMα, which are M2 macrophage differentiation markers. Intriguingly, the glycolysis inhibitor 2-DG suppresses mitochondrial OXPHOS and impairs JAK/STAT6 pathway signaling to inhibit M2 differentiation [130].

Neutrophils may mediate immune suppression in the TME. C-Kit is the receptor of stem cell factor (SCF), which is a marker of neutrophil immaturity. The 4T1 mammary tumors express SCF and promote OXPHOS activity in neutrophils via SCF-c-Kit signaling. Tumor-elicited neutrophils upregulate the expression of Complexes I and IV, impairing the function of tumor-infiltrating T cells [131].

## 6. OXPHOS Is a Novel Therapeutic Target That Can Be Leveraged to Overcome Cancer Drug Resistance

### 6.1. OXPHOS Inhibitors in Cancer Therapy

Targeting OXPHOS in combination with standard therapy can specifically eradicate resistant cells and prolong the survival time of cancer patients with certain cancers [16,18,132,133] (Table 1). OPB-51602 is a novel OXPHOS inhibitor that can repress Complex I activity to diminish mitochondrial OXPHOS. OPB-51602 resensitizes patients who develop resistance to EGFR TKI, resulting in tumor regression and profound metabolic responses [22]. Imatinib therapy contributes to the enhancement of OXPHOS activity and this metabolic phenotype may lead to imatinib resistance in gastrointestinal stromal tumors (GISTs). Targeting OXPHOS with the mitochondrial OXPHOS inhibitor VLX600 is an effective strategy for counteracting imatinib resistance. VLX600 decreases the oxygen consumption rate (OCR) in HCT116 cells and inhibits the activities of Complex I, II and IV, leading to mitochondrial dysfunction and glucose dependence. VLX600 targets quiescent cells in colon cancer, which significantly increases chemotherapy efficacy [21,134]. Mubritinib is a selective ERBB2 inhibitor with an anticancer effect in bladder, kidney and prostate cancer [135]. A recent study finds that mubritinib displays anti-leukemic activity by inhibiting NADH dehydrogenase activity. Chemotherapy-resistant AML cells with high OXPHOS is sensitive to mubritinib, which is associated with poor outcome [136].

OXPHOS complexes are embedded in the mitochondrial inner membrane. The mitochondrial outer membrane affects apoptosis by regulating the BCL-2 family of proteins. Dual inhibition of OXPHOS and BCL-2 can delay the generation of drug resistance. BAM15 is a mitochondrial uncoupling agent targeting OXPHOS. ABT737 is a BCL-2 inhibitor. The combination of BAM15 and ABT737 promotes melanoma cell apoptosis induced by MAPK pathway inhibitors [137]. OXPHOS inhibitors selectively eliminate CSCs but do not affect normal cells, improving the efficacy of anticancer drugs. High levels of aldehyde dehydrogenase (ALDH) promote chemotherapy resistance and enhance cancer stemness. ALDH1A family selective inhibitors (ALDH1Ai) target ovarian CSCs. ALDH1Ai upregulate UCPs and inhibit OXPHOS activity to mediate necroptosis in ovarian CSCs [138].

### 6.2. Novel Potential Biomarkers to Enhance the Application of OXPHOS Inhibitors

While OXPHOS is a significant target for cancer therapy, it is hard to predict the efficacy of OXPHOS inhibitors. Mitochondrial gene expression and TNBC type can predict the efficacy of OXPHOS inhibitor IACS-010759. On the other hand, IACS-010759 resistance in TNBC is associated with epithelial mesenchymal transition (EMT) signature and increased expression of *AXL*, a member of the TAM family. IACS-010759 delays the resistance to CDK4/6 inhibitor palbociclib in TNBC [139].

Genomic instability is related to tumorigenesis [140]. Homologous recombination-defective (HRD) cancers are dependent on OXPHOS to supply NAD and ATP for the DNA repair pathway. HRD cancer cells undergo metabolic reprogramming, including enhanced OXPHOS and decreased glycolysis. OXPHOS activity confers metformin sensitivity to HRD cancer cells and is associated with the efficacy of PARP inhibitors [141]. The mtDNA mutations in some human tumors may contribute to cell proliferation advantages during tumorigenesis [8]. Mutations in the mitochondrial Complex I gene enable cancer cells to suppress OXPHOS upregulation but sensitize them to Complex I inhibitor phenformin when in low-glucose state [142]. The capacity of glucose utilization clearly affects sensitivity to the OXPHOS inhibitor in cancer cells. Glucose transporter GLUT1 expression induces resistance to OXPHOS inhibitors [143]. Similarly, overexpression of GLUT3 significantly minimizes phenformin sensitivity in a low-glucose state [142]. On the other hand, inhibition of protein synthesis can enhance OXPHOS inhibitor resistance [143]. Hence, mtDNA mutations, glucose uptake capacity and protein synthesis rate may be novel potential biomarkers that can be used to enhance the application of OXPHOS inhibitors in cancer therapy.

**Table 1:** A list of drugs related to OXPHOS systems for cancer therapy.

Therapeutic Agent	Clinical Testing Phase	Observations	References
Inhibition of Complex I			
IACS-010759	Preclinical	IACS-010759 is a Complex I inhibitor and mediates apoptosis in AML dependent on OXPHOS; IACS-010759 in combination with radiotherapy sensitized the PD-1-resistant model to anti-PD-1 agents and prolonged survival time.	[117,118]
Phenformin	Preclinical	Mutations in the mitochondrial Complex I gene enable cancer cells to suppress OXPHOS upregulation but sensitize them to Complex I inhibitor phenformin when in low-glucose state.	[142]
Metformin	Several hundred trials in progress	Metformin increases intracellular ROS levels and induces apoptosis in pancreatic CSCs.	[100]
Mubritinib	Preclinical	Mubritinib displays anti-leukemic activity by inhibiting NADH dehydrogenase activity.	[136]
OPB-51602	Phase I	OPB-51602 resensitizes patients who develop resistance to EGFR TKI, resulting in tumor regression and profound metabolic responses.	[22]
Inhibition of Complex II			
Lonidamine	Phase III	Lonidamine promotes anti-tumor activity of conventional cytotoxic drugs in NSCLC.	[121]
Inhibition of Complex III			
Atovaquone	Preclinical	Atovaquone has anti-tumor activity and significantly eliminates CSCs in breast cancer cells.	[19]
Inhibition of Complex IV			
Arsenic trioxide	Clinical use for APL	Arsenic trioxide acutely upregulates oxygen consumption and sensitizes tumors to radiotherapy.	[23]
Inhibition of Complex V			
Oligomycin	Preclinical	Oligomycin specifically eliminates pancreatic CSCs resistant to KRAS inhibitors.	[20]
Gboxin	Preclinical	Gboxin sensitizes HCC to metformin by abrogating the interaction between TOMM34 and ATP5B.	[101]

Abbreviations: ROS, reactive oxygen species; CSCs, cancer stem cells; TKI, tyrosine kinase inhibitor; NSCLC, non-small cell lung cancer; APL, acute promyelocytic leukemia; HCC, hepatocellular carcinoma.

## 7. Conclusions

Recent studies have demonstrated that resistance to anticancer drugs depends on OXPHOS in various cancers and that this resistance can be reversed by inhibiting mitochondrial OXPHOS. Resistant cancer cells show activation of a variety of oncogenic pathways that promote OXPHOS activity and cell survival after treatment with conventional therapies. CSCs may be enriched among a minimal residual cancer cell population after exposure to cytotoxic agents, leading to cancer recurrence. The metabolic signature of CSCs is increased OXPHOS, which contributes to their insensitivity to cancer treatment. The effect of oxidative metabolism differs among immune cells and it plays a dual role in immunotherapy resistance. Downregulation of OXPHOS impairs the function of tumor-infiltrating T cells and diminishes antitumor immune responses to immune checkpoint therapy. OXPHOS inhibitors have significantly prevented or delayed cancer drug resistance and sensitize resistant cancer cells to standard therapy in preclinical research. However, the efficacy of OXPHOS inhibitors varies dramatically in different cancers. Sensitivity to OXPHOS inhibitors is associated with complex factors, such as mtDNA mutations and glycolysis, which need to be further explored to improve treatment responses. Strategies to specifically target cancer cells and decrease their negative effects on immune cells are important for increasing the application of OXPHOS inhibitors in clinical practice.
